# *NOTCH1*: Review of its role in lymphatic development and study of seven families with rare pathogenic variants

**DOI:** 10.1002/mgg3.1529

**Published:** 2020-11-28

**Authors:** Sandro Michelini, Maurizio Ricci, Roberta Serrani, Shila Barati, Sercan Kenanoglu, Dominika Veselenyiova, Danjela Kurti, Mirko Baglivo, Syed Hussain Basha, Sasi Priya, Astrit Dautaj, Munis Dundar, Juraj Krajcovic, Matteo Bertelli

**Affiliations:** ^1^ Department of Vascular Rehabilitation San Giovanni Battista Hospital Rome Italy; ^2^ Division of Rehabilitation Medicine Azienda Ospedaliero‐Universitaria Ospedali Riuniti di Ancona Ancona Italy; ^3^ MAGI EUREGIO Bolzano Italy; ^4^ Department of Medical Genetics Faculty of Medicine Erciyes University Kayseri Turkey; ^5^ Department of Biology Faculty of Natural Sciences University of Ss. Cyril and Methodius in Trnava Trnava Slovakia; ^6^ MAGI‐Balkans Tirana Albania; ^7^ Innovative Informatica Technologies Hyderabad India; ^8^ EBTNA‐LAB Rovereto Italy; ^9^ MAGI'S LAB Rovereto Italy

**Keywords:** genetic diagnosis, lymphedema, Next Generation Sequencing (NGS), *NOTCH1*

## Abstract

**Background:**

We developed a Next‐Generation‐Sequencing (NGS) protocol to screen the most frequent genetic variants related to lymphedema and a group of candidate genes. The aim of the study was to find the genetic cause of lymphedema in the analyzed patients.

**Methods:**

We sequenced a cohort of 246 Italian patients with lymphatic malformations. In the first step, we analyzed genes known to be linked to lymphedema: 235 out of 246 patients tested negative for the most frequent variants and underwent testing for variants in a group of candidate genes, including the *NOTCH1* gene, selected from the database of mouse models. We also performed in silico analysis to observe molecular interactions between the wild‐type and the variant amino acids and other protein residues.

**Results:**

Seven out of 235 probands, five with sporadic and two with familial lymphedema, were found to carry rare missense variants in the *NOTCH1* gene.

**Conclusions:**

Our results propose that *NOTCH1* could be a novel candidate for genetic predisposition to lymphedema.

## Graphical abstract

Background: We developed a Next‐Generation‐Sequencing (NGS) protocol to screen the most frequent genetic variants related to lymphedema, including a group of candidate genes.Methods and Results: We sequenced a cohort of 246 Italian patients with lymphatic malformations. In the first step, we analyzed genes known to be linked to lymphedema: 235 out of 246 patients tested negative for the most frequent variants and underwent testing for variants in a group of candidate genes, including the NOTCH1 gene, selected from the database of mouse models. The aim of the study was to define the genotype of these patients, in order to explore the role of the candidate gene NOTCH1 in lymphedema.We studied lymphatic malformations related to NOTCH1 variants. We also performed in silico analysis to observe molecular interactions between the wild‐type and the variant amino acids and other protein residues. Seven out of 235 probands, five with sporadic and two with familial lymphedema, were found to carry rare missense variants in the NOTCH1 gene.Conclusions: Our results propose that NOTCH1 could be a novel candidate for genetic predisposition to lymphedema.

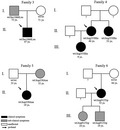

## 1 INTRODUCTION

The *NOTCH1* gene (OMIM: *190198) encodes a member of the NOTCH family of type I transmembrane proteins. Members of this protein family share structural characteristics, including an extracellular domain consisting of multiple epidermal growth factor‐like (EGF) repeats, and an intracellular region consisting of many different domain types.

NOTCH proteins are single‐pass transmembrane receptors that regulate cell fate during development. The Notch family includes four receptors, *NOTCH1*, *NOTCH2* (OMIM: *600275), *NOTCH3* (OMIM: *600276), and *NOTCH4* (OMIM: *164951), whose ligands include *JAG1* (OMIM: *601920), *JAG2* (OMIM: *602570), *DLL1* (OMIM: *606582), *DLL3* (OMIM: *602768) and *DLL4* (OMIM: *605185). All the receptors have an extracellular domain containing multiple epidermal growth factor‐like repeats and an intracellular region containing the RAM domain, ankyrin repeats, and a C‐terminal PEST domain.

Notch signaling is an intercellular signaling pathway, conserved through evolution, that regulates interactions between physically adjacent cells through the binding of Notch family receptors to their cognate ligands. The preproprotein encoded is processed proteolytically in the trans‐Golgi network to generate two polypeptide chains that heterodimerize to form the mature cell‐surface receptor. This receptor plays a role in the development of many types of cells and tissues.

Fatima et al. (2014), Zheng et al. (2011), Niessen et al. (2011), and others demonstrated that lymphatic endothelial cells (LECs) show *NOTCH* pathway activity and that Notch1 is a key regulator of LEC sprouting and growth during the morphogenesis of lymphatic vessels in the developing mouse embryo. Interestingly, the Notch pathway negatively regulates lymphatic sprouting and directs stalk cell specification in LECs (Fatima et al., 2014; Niessen et al., 2011; Zheng et al., 2011). These authors showed that the inhibition of Notch1 and DLL4, with specific function‐blocking antibodies, results in defective postnatal lymphatic development in mice. In a mammalian system, they demonstrated that the Notch1‐DLL4 signaling pathway regulates postnatal lymphatic development and pathological lymphangiogenesis. Notch1 plays also a fundamental role in valve morphogenesis (Murtomaki et al., 2014).

The transmembrane receptor Notch1 is a critical regulator of arterial differentiation and blood vessel sprouting. Functional blockade of Notch1 and its ligand DLL4 leads to postnatal lymphatic defects in mice. Moreover, LTB4 biosynthesis was elevated in lymphedema patients. Interestingly, *Notch1*‐/‐ mice were found resistant to the benevolent effects of LTB4 antagonism (Tian et al., 2017). Fatima et al. (2014) demonstrated that Notch1 is a key regulator of LEC sprouting and growth during the morphogenesis of lymphatic vessels in the developing mouse embryo. Conditional LEC‐specific deletion of Notch1 in mice resulted in significant lymphatic overgrowth with dilated lymphatic vessels, and Notch1‐mutant LECs exhibited increased proliferation, decreased cell death, and enhanced sprouting (Fatima et al., 2014). Notch signaling interacts with the VEGF pathway to regulate blood vessel sprouting by selecting tip and stalk cells.

VEGF‐C and VEGF‐D, acting through VEGF receptor 3, are key inducers of lymphangiogenesis. Loss of Vegfc leads to complete aplasia of the lymphatic vessels and embryo death due to edema, whereas VEGF‐D is indispensable for lymphatic development in mice.

Zheng et al. (2011) demonstrated NOTCH pathway activity in LECs and NOTCH target genes on stimulation with VEGF or VEGF‐C. These results indicate that the Notch pathway controls lymphatic endothelial quiescence, and explain why LECs respond less to VEGF than to VEGF‐C.

Pathogenic variants in *NOTCH1* are associated with aortic valve disease (OMIM: #109730) and with Adams‐Oliver syndrome (OMIM: #616028), both with autosomal dominant inheritance. Garg et al. (2005) showed that mutations in the signaling and transcriptional regulator NOTCH1 cause a spectrum of developmental aortic valve anomalies (bicuspid) and severe valve calcification (AOVD1; OMIM: #109730) in nonsyndromic autosomal dominant human pedigrees (Garg et al., 2005). Furthermore, lymphatic abnormalities and lymphedema are rare manifestations of Adams‐Oliver syndrome (Amor et al., 2000).

Variants in the *NOTCH1* gene are also associated with a predisposition for the nonsyndromic tetralogy of Fallot (TOF). Variants in this gene are found in almost 7% of TOF patients (Page et al., 2019).

We felt the need to look for new candidate genes in order to explain the phenotype of our patients. Here we focused on *NOTCH1*, believing it could be a good candidate, as suggested by many studies on mouse models.

## 2 MATERIALS AND METHODS

### 2.1 Ethical compliance

The study was approved by an ethics committee.

### 2.2 Clinical evaluation

We retrospectively enrolled 246 Caucasian patients diagnosed with lymphedema in hospitals across Italy. No consanguinity was reported in their families. Clinical diagnosis of lymphedema was made according to generally accepted criteria. Genetic testing was performed on germline DNA extracted from saliva or peripheral blood of probands. Segregation analysis was performed using DNA extracted from the saliva of probands’ relatives.

### 2.3 Genetic analysis

A custom‐made oligonucleotide probe library was designed to capture all coding exons and flanking exon/intron boundaries (±15 bp) of 29 genes known to be associated with lymphedema (Michelini et al., 2018). We added the candidate gene NOTCH1 to our panel. DNA from probands was analyzed for genetic variants. Variants with likely clinical significance were confirmed by bidirectional Sanger sequencing on a CEQ8800 Sequencer (Beckman Coulter). Segregation in available family members was performed for variants identified in probands harboring heterozygous *NOTCH1* variants by Sanger sequencing.

We developed a Next‐Generation‐Sequencing (NGS) protocol for the screening of the most frequent genetic variants, namely *ADAMTS3* (NG_046955.1; OMIM: *605011), *CELSR1* (NG_030466.2; OMIM: *604523), *EPHB4* (NG_052671.1; OMIM: *600011), *FAT4* (NG_033865.1; OMIM: *612411), *FLT4* (NG_011536.1; OMIM: *136352), *FOXC2* (NG_012025.2; OMIM: *602402), *GATA2* (NG_029334.1; OMIM: *137295), *GJA1* (NG_008308.1; OMIM: *121014), *GJC2* (NG_011838.1; OMIM: *608803), *HGF* (NG_016274.2; OMIM: *142409), *KIF11* (NG_032580.1; OMIM: *148760), *PIEZO1* (NG_042229.1; OMIM: *611184), *PTPN14* (NG_028036.1; OMIM: *603155), *SOX18* (NG_008095.1; OMIM: *601618) and *VEGFC* (NG_034216.1; OMIM: *601528), adding the candidate gene *NOTCH1* (NG_007458.1).

We searched the international databases dbSNP and Human Gene Mutation Database professional for all nucleotide changes. In silico evaluation of the pathogenicity of sequence changes in NOTCH1 was performed using the Variant Effect Predictor tool and MutationTaster. Minor allele frequencies were checked in the Genome Aggregation Database (gnomAD). All variants were evaluated according to the American College of Medical Genetics and Genomics guidelines (Richards et al., 2015). Detailed pre‐test genetic counseling was provided to all subjects, who were then invited to sign informed consent to use of their anonymized genetic results for research.

### 2.4 In silico analysis

The primary amino acid sequences of NOTCH1 in FASTA format (Tables 1 and 2) were used as targets to search the Swiss Model template library (SMTL) version 2019‐10‐24 and Protein Data Bank (PDB) release 2019‐10‐18 (Berman et al., 2000) for matching evolution‐related structures by means of BLAST (Basic Local Alignment Search Tool) (Camacho et al., 2009) and HHBlits (Remmert et al., 2012). Models were based on target‐template alignment using ProMod3 of the Swiss‐Model server (Waterhouse et al., 2018). Coordinates conserved between the target and the template were copied from the template to the model. Insertions and deletions were remodeled using a fragment library. Side chains were then rebuilt. Finally, the geometry of the resulting model was regularized with the CHARMM27 force field (Mackerell et al., 2004). In the case of failure of loop modeling with ProMod3, an alternative model was built with ProMod‐II (Guex et al., 2009). Global and per‐residue model quality was assessed using the QMEAN scoring function (Benkert et al., 2011). BIOVIA Discovery Studio Visualizer ver17.2 (Studio, 2016) was used to visualize the modeled protein, to vary the targeted amino acids, and to analyze interactions at the molecular level.

**TABLE 1:** Primary amino acid sequence used to search for templates in order to build models for NOTCH1

MPPLLAPLLCLALLPALAARGPRCSQPGETCLNGGKCEAANGTEACVCGGAFVGPRCQDPNPCLSTPCKNAGTCHVVDRRGVADYACSCALGFSGPLCLTPLDNACLTNPCRNGGTCDLLTLTEYKCRCPPGWSGKSCQQADPCASNPCANGGQCLPFEASYICHCPPSFHGPTCRQDVNECGQKPGLCRHGGTCHNEVGSYRCVCRATHTGPNCERPYVPCSPSPCQNGGTCRPTGDVTHECACLPGFTGQNCEENIDDCPGNNCKNGGACVDGVNTYNCRCPPEWTGQYCTEDVDECQLMPNACQNGGTCHNTHGGYNCVCVNGWTGEDCSENIDDCASAACFHGATCHDRVASFYCECPHGRTGLLCHLNDACISNPCNEGSNCDTNPVNGKAICTCPSGYTGPACSQDVDECSLGANPCEHAGKCINTLGSFECQCLQGYTGPRCEIDVNECVSNPCQNDATCLDQIGEFQCICMPGYEGVHCEVNTDECASSPCLHNGRCLDKINEFQCECPTGFTGHLCQYDVDECASTPCKNGAKCLDGPNTYTCVCTEGYTGTHCEVDIDECDPDPCHYGSCKDGVATFTCLCRPGYTGHHCETNINECSSQPCRHGGTCQDRDNAYLCFCLKGTTGPNCEINLDDCASSPCDSGTCLDKIDGYECACEPGYTGSMCNINIDECAGNPCHNGGTCEDGINGFTCRCPEGYHDPTCLSEVNECNSNPCVHGACRDSLNGYKCDCDPGWSGTNCDINNNECESNPCVNGGTCKDMTSGYVCTCREGFSGPNCQTNINECASNPCLNQGTCIDDVAGYKCNCLLPYTGATCEVVLAPCAPSPCRNGGECRQSEDYESFSCVCPTGWQGQTCEVDINECVLSPCRHGASCQNTHGGYRCHCQAGYSGRNCETDIDDCRPNPCHNGGSCTDGINTAFCDCLPGFRGTFCEEDINECASDPCRNGANCTDCVDSYTCTCPAGFSGIHCENNTPDCTESSCFNGGTCVDGINSFTCLCPPGFTGSYCQHDVNECDSQPCLHGGTCQDGCGSYRCTCPQGYTGPNCQNLVHWCDSSPCKNGGKCWQTHTQYRCECPSGWTGLYCDVPSVSCEVAAQRQGVDVARLCQHGGLCVDAGNTHHCRCQAGYTGSYCEDLVDECSPSPCQNGATCTDYLGGYSCKCVAGYHGVNCSEEIDECLSHPCQNGGTCLDLPNTYKCSCPRGTQGVHCEINVDDCNPPVDPVSRSPKCFNNGTCVDQVGGYSCTCPPGFVGERCEGDVNECLSNPCDARGTQNCVQRVNDFHCECRAGHTGRRCESVINGCKGKPCKNGGTCAVASNTARGFICKCPAGFEGATCENDARTCGSLRCLNGGTCISGPRSPTCLCLGPFTGPECQFPASSPCLGGNPCYNQGTCEPTSESPFYRCLCPAKFNGLLCHILDYSFGGGAGRDIPPPLIEEACELPECQEDAGNKVCSLQCNNHACGWDGGDCSLNFNDPWKNCTQSLQCWKYFSDGHCDSQCNSAGCLFDGFDCQRAEGQCNPLYDQYCKDHFSDGHCDQGCNSAECEWDGLDCAEHVPERLAAGTLVVVVLMPPEQLRNSSFHFLRELSRVLHTNVVFKRDAHGQQMIFPYYGREEELRKHPIKRAAEGWAAPDALLGQVKASLLPGGSEGGRRRRELDPMDVRGSIVYLEIDNRQCVQASSQCFQSATDVAAFLGALASLGSLNIPYKIEAVQSETVEPPPPAQLHFMYVAAAAFVLLFFVGCGVLLSRKRRRQHGQLWFPEGFKVSEASKKKRREPLGEDSVGLKPLKNASDGALMDDNQNEWGDEDLETKKFRFEEPVVLPDLDDQTDHRQWTQQHLDAADLRMSAMAPTPPQGEVDADCMDVNVRGPDGFTPLMIASCSGGGLETGNSEEEEDAPAVISDFIYQGASLHNQTDRTGETALHLAARYSRSDAAKRLLEASADANIQDNMGRTPLHAAVSADAQGVFQILIRNRATDLDARMHDGTTPLILAARLAVEGMLEDLINSHADVNAVDDLGKSALHWAAAVNNVDAAVVLLKNGANKDMQNNREETPLFLAAREGSYETAKVLLDHFANRDITDHMDRLPRDIAQERMHHDIVRLLDEYNLVRSPQLHGAPLGGTPTLSPPLCSPNGYLGSLKPGVQGKKVRKPSSKGLACGSKEAKDLKARRKKSQDGKGCLLDSSGMLSPVDSLESPHGYLSDVASPPLLPSPFQQSPSVPLNHLPGMPDTHLGIGHLNVAAKPEMAALGGGGRLAFETGPPRLSHLPVASGTSTVLGSSSGGALNFTVGG

**TABLE 2:** Top 10 models for the 3D modeling of NOTCH1 structure

Template	Seq identity	Oligo‐state	QSQE	Found by	Method	Resolution	Seq similarity	Coverage	Description
6py8.2.E	100.00	Monomer	–	BLAST	X‐ray	3.75A°	0.61	0.14	Neurogenic locus notch homolog protein 1
6py8.1.E	100.00	Monomer	–	BLAST	X‐ray	3.75 Å	0.61	0.14	Neurogenic locus notch homolog protein 1
3eto.1.A	97.50	Monomer	–	BLAST	X‐ray	2.00 Å	0.64	0.09	Neurogenic locus notch homolog protein 1
3195.1.C	100.00	Homo‐dimer	0.37	BLAST	X‐ray	2.19 Å	0.63	0.09	Neurogenic locus notch homolog protein 1
3195.1.E	100.00	Homo‐dimer	0.37	BLAST	X‐ray	2.19 Å	0.64	0.09	Neurogenic locus notch homolog protein 1
2f8y.1.A	100.00	Monomer	–	BLAST	X‐ray	1.55 Å	0.60	0.10	Notch homolog 1, translocation associated (Drosophila)
3v79.1.A	100.00	Monomer	–	BLAST	X‐ray	3.85 Å	0.60	0.09	Neurogenic locus notch homolog protein 1
2f8y.2.A	100.00	Monomer	–	BLAST	X‐ray	1.55 Å	0.60	0.09	Notch homolog 1, translocation associated (Drosophila)
5uk5.1.A	96.91	Monomer	–	BLAST	X‐ray	2.51 Å	0.65	0.08	Neurogenic locus notch homolog protein 1
1yyh.1.A	100.00	Monomer	–	BLAST	X‐ray	1.90 Å	0.60	0.10	Notch1, ankyrin domain

## 3 RESULTS

### 3.1 Clinical and genetic evaluation

**FIGURE 1:**
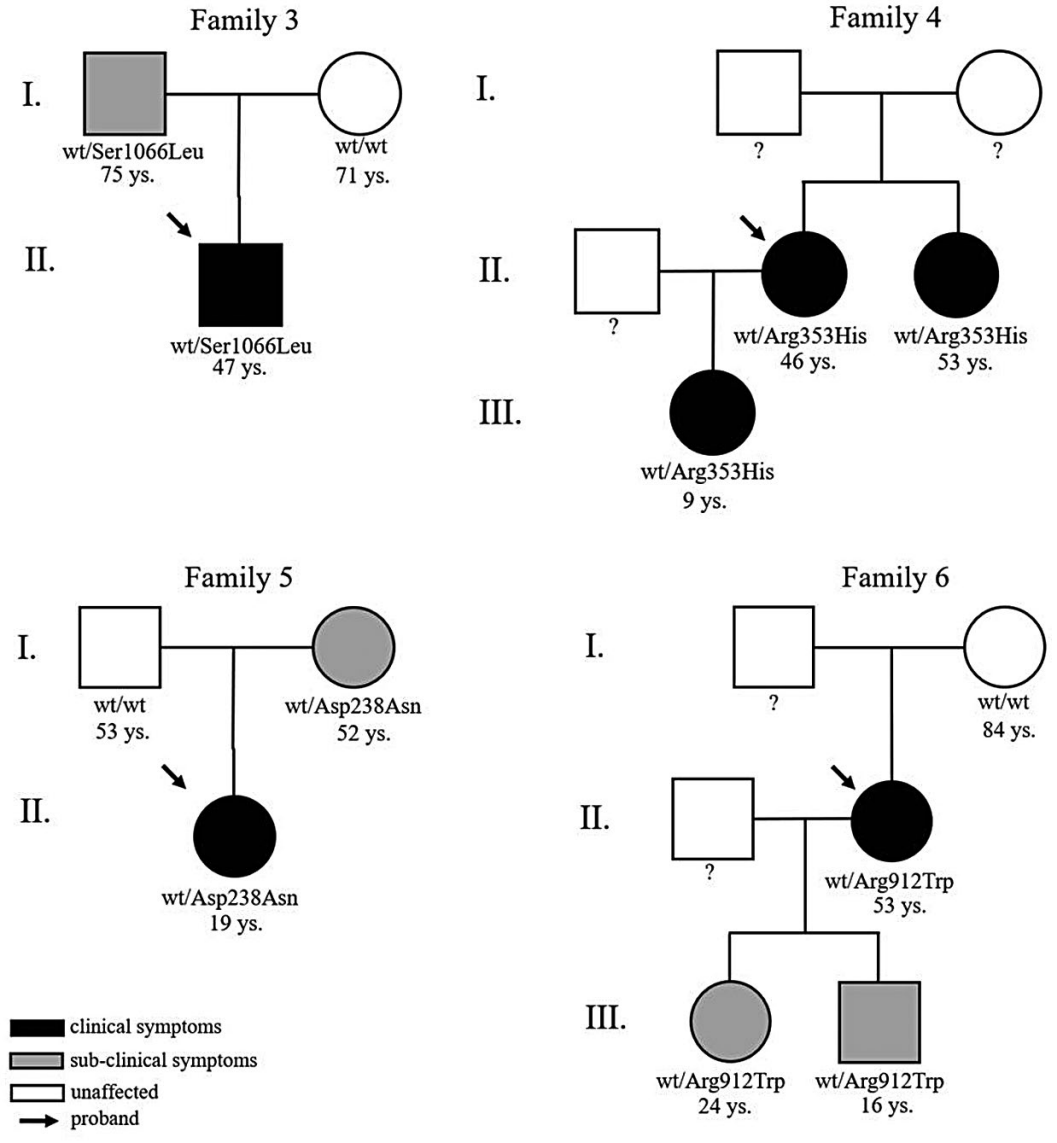
Pedigree of families which segregate reported variant in *NOTCH1*

Out of 235 lymphedema patients tested for variants in *NOTCH1*, seven were found to carry rare variants in this gene. The mean age of probands was 39.2 years and the male/female ratio was 0.28. The mean age at diagnosis was 12.42 years. Five out of seven cases were sporadic. The clinical features are shown in Table 3; the segregation of the variant in the families of the probands is shown in Figure 1.

**TABLE 3:** Clinical features of patients and their parents with rare variants in *NOTCH1*

Family	Pedigree	Sex	Age in years	Clinical features	Age at onset	Affected relatives	Variant nomenclature
1	Proband	F	49	Edema of lower limbs and lipedema	14 years	No	*NOTCH1*:NM_017617.4:c.6395C>T: NP_060087.3:p.(Thr2132Met)
2	Proband	M	16	Edema of lower limbs and scrotum	9 months	No	*NOTCH1*:NM_017617.4:c.3954 T > A: NP_060087.3:p.(Asn1318Lys)
3	Proband	M	47	Edema of right lower limb	10 years	Father	*NOTCH1*:NM_017617.4:c.3197C>T: NP_060087.3:p.(Ser1066Leu)
3	Father	M	75	Healthy	/	Father	*NOTCH1*:NM_017617.4:c.3197C>T: NP_060087.3:p.(Ser1066Leu)
4	Proband	F	45	Edema of lower limbs from knee to ankle; edema of hands	9 years	sister and daughter	*NOTCH1*:NM_017617.4:c.1058G>A: NP_060087.3:p.(Arg353His)
4	Sister	F	53	Right lower limb edema after lymphangitis	30 years	sister and daughter	*NOTCH1*:NM_017617.4:c.1058G>A: NP_060087.3:p.(Arg353His)
4	Daughter	F	9	Right foot edema after acute tonsillitis	8 years	sister and daughter	*NOTCH1*:NM_017617.4:c.1058G>A: NP_060087.3:p.(Arg353His)
5	Proband	F	18	Edema of right lower limb	13 years	Mother	*NOTCH1*:NM_017617.4:c.712G>A: NP_060087.3:p.(Asp238Asn)
5	Mother	F	52	Healthy	/	Mother	*NOTCH1*:NM_017617.4:c.712G>A: NP_060087.3:p.(Asp238Asn)
6	Proband	F	52	Edema of left lower limb	23 years	Daughter and Son	*NOTCH1*:NM_017617.4:c.2734C>T: NP_060087.3:p.(Arg912Trp)
6	Daughter	F	24	Healthy	/	Daughter and Son	*NOTCH1*:NM_017617.4:c.2734C>T: NP_060087.3:p.(Arg912Trp)
6	Son	M	16	Healthy	/	Daughter and Son	*NOTCH1*:NM_017617.4:c.2734C>T: NP_060087.3:p.(Arg912Trp)
7	Proband	F	48	Edema of right lower limb edema from knee to foot	17 years	No	*NOTCH1*:NM_017617.4:c.c.2353G>A: NP_060087.3:p.(Gly785Ser)

In the first family, the proband (female, 49 years) has sporadic edema of the lower limbs and lipedema with onset at age 14 years. The proband carries the missense variant *NOTCH1*:NM_017617.4:c.6395C>T: NP_060087.3:p.(Thr2132Met) which involves a poorly conserved residue. The prediction databases Mutation Taster, Sift, and PolyPhen‐2 classify it as *polymorphism*, *tolerated*, and *possibly damaging*, respectively. Amino acid substitution causes a potential loss of the TOPO_DOM cytoplasmic domain. The dbSNP ID of the variant is rs777501680 and the frequency in the GnomAD database is 0.00001.

In the second family, the proband (male, 16 years) has sporadic edema of the lower limbs and scrotum with onset at age 9 months. The proband carries the missense variant *NOTCH1*:NM_017617.4:c.3954 T > A: NP_060087.3:p.(Asn1318Lys) which involves a highly conserved protein residue. Prediction databases Mutation Taster, Sift, and PolyPhen‐2 classify it as *disease‐causing, deleterious*, and *probably* *damaging*, respectively. Amino acid substitution causes loss of the EGF‐like 34 domain. The dbSNP ID of the variant is rs754634957 and the frequency in the GnomAD database is 0.00002.

In the third family, the proband (male, 47 years) has sporadic edema of the right lower limb with onset at age 10. The proband carries the missense variant *NOTCH1*:NM_017617.4:c.3197C>T: NP_060087.3:p.(Ser1066Leu) which involves a highly conserved protein residue. Prediction databases Mutation Taster, Sift, and PolyPhen‐2 classify it as *disease‐causing, deleterious*, and *probably damaging*, respectively. Amino acid substitution causes loss of the EGF‐like 28 domain. The dbSNP ID of the variant is rs771739312 and the frequency in the GnomAD database is 0.00001. The variant was inherited from the healthy father, he reported episodes of intermittent edema of lower limbs.

In the fourth family, the proband (female, 45 years) has familial edema of the lower limbs (from knee to ankle) and hands with onset at age 9. The proband has a daughter and a sister, both affected. The proband carries the missense variant *NOTCH1*:NM_017617.4:c.1058G>A: NP_060087.3:p.(Arg353His) which involves a highly conserved protein residue. Prediction databases Mutation Taster, Sift, and PolyPhen‐2 classify it as *disease‐causing*, *deleterious*, and *probably damaging*, respectively. Amino acid substitution causes loss of the EGF‐like 9 domain and possibly the calcium‐binding domain. The dbSNP ID of the variant is rs750215904 and the frequency in the GnomAD database is 0.000007. Sister and daughter carry the same variant of the proband and they reported episodes of edema after lymphangitis and tonsillitis, respectively.

In the fifth family, the proband (female, 18 years) has sporadic edema of the right lower limb with onset at age 13. The proband carries the missense variant *NOTCH1*:NM_017617.4:c.712G>A: NP_060087.3:p.(Asp238Asn) which involves a highly conserved residue. Prediction databases Mutation Taster, Sift, and PolyPhen‐2 classify it as *disease‐causing*, *tolerated* and *possibly damaging*, respectively. Amino acid substitution causes loss of the EGF‐like 6 domain. The dbSNP ID of the variant is rs550554578 and the frequency in the GnomAD database is 0.000007. The mother carries the same variant as the proband, unlike the father. Subclinical analysis reported that she has episodes of cyclic edema in the lower limbs.

In the sixth family, the proband (female, 52 years) has sporadic edema of the left lower limb with onset at age 23. The proband carries the missense variant *NOTCH1*:NM_017617.4:c.2734C>T: NP_060087.3:p.(Arg912Trp) which involves a partially conserved residue. Prediction databases Mutation Taster, Sift, and PolyPhen‐2 classify it as *disease‐causing*, *deleterious*, and *probably damaging*, respectively. Amino acid substitution causes loss of the EGF‐like 24 domain. The dbSNP ID of the variant is rs201620358 and the frequency in the GnomAD database is 0.001 (Tables 3 and 4). Proband's daughter and son carries the same variant. Their subclinical exams reported intermittent edema of the lower limbs.

**TABLE 4:** Variants features and predictions

Variant	dbSNPid	Mutation taster	Affected domains	Sift	PolyPhen‐2	Frequency
*NOTCH1*:NM_017617.4:c.6395C>T: NP_060087.3:p.(Thr2132Met)	rs777501680	Prediction: polymorphism; poorly conserved residue	TOPO_DOM cytoplasmic (Potential)	Tolerated	Possibly damaging	0.00001
*NOTCH1*:NM_017617.4:c.3954 T > A: NP_060087.3:p.(Asn1318Lys)	rs754634957	Prediction: Disease‐causing; highly conserved residue	EGF‐like 34	Deleterious	Probably damaging	0.00002
*NOTCH1*:NM_017617.4:c.3197C>T: NP_060087.3:p.(Ser1066Leu)	rs771739312	Prediction: Disease‐causing; highly conserved residue	EGF‐like 28	Deleterious	Possibly damaging	0.00001
*NOTCH1*:NM_017617.4:c.1058G>A: NP_060087.3:p.(Arg353His)	rs750215904	Prediction: Disease‐causing; highly conserved residue	EGF‐like 9; calcium‐binding (Potential)	Deleterious	Probably damaging	0.000007
*NOTCH1*:NM_017617.4:c.712G>A: NP_060087.3:p.(Asp238Asn)	rs550554578	Prediction: Disease‐causing; highly conserved residue	EGF‐like 6	Tolerated	Possibly damaging	0.000007
*NOTCH1*:NM_017617.4:c.2734C>T: NP_060087.3:p.(Arg912Trp)	rs201620358	Prediction: Disease‐causing; partially conserved residue	EGF‐like 24	Deleterious	Possibly damaging	0.001
*NOTCH1*:NM_017617.4:c.c.2353G>A: NP_060087.3:p.(Gly785Ser)	rs764191723	Prediction: Disease‐causing; highly conserved residue	EGF‐like 20	Deleterious	Probably damaging	0.00008

In the seventh family, the proband (female, 48 years) has right lower limb edema (from knee to foot) with onset at age 17. A cousin of the mother is affected. The proband carries the missense variant *NOTCH1*:NM_017617.4:c.c.2353G>A: NP_060087.3:p.(Gly785Ser) which involves a highly conserved protein residue. Prediction databases Mutation Taster, Sift, and PolyPhen‐2 classify it as *disease‐causing*, *deleterious*, and *probably damaging*, respectively. Amino acid substitution causes loss of the EGF‐like 20 domain. The dbSNP ID of the variant is rs764191723 and the frequency in the GnomAD database is 0.00008.

### 3.2 In silico analysis, template selection, and model building

Template search with BLAST and HHBlits was performed against the Swiss‐Model template library (SMTL, last update: 2019‐10‐24, last included PDB release: 2019‐10‐18). The target sequence was searched against the primary amino acid sequence contained in the SMTL. A total of 628 templates, matching with varied sequence identity and quality percentages, were found. Details of the top 10 templates are shown in Table 2.

**FIGURE 2:**
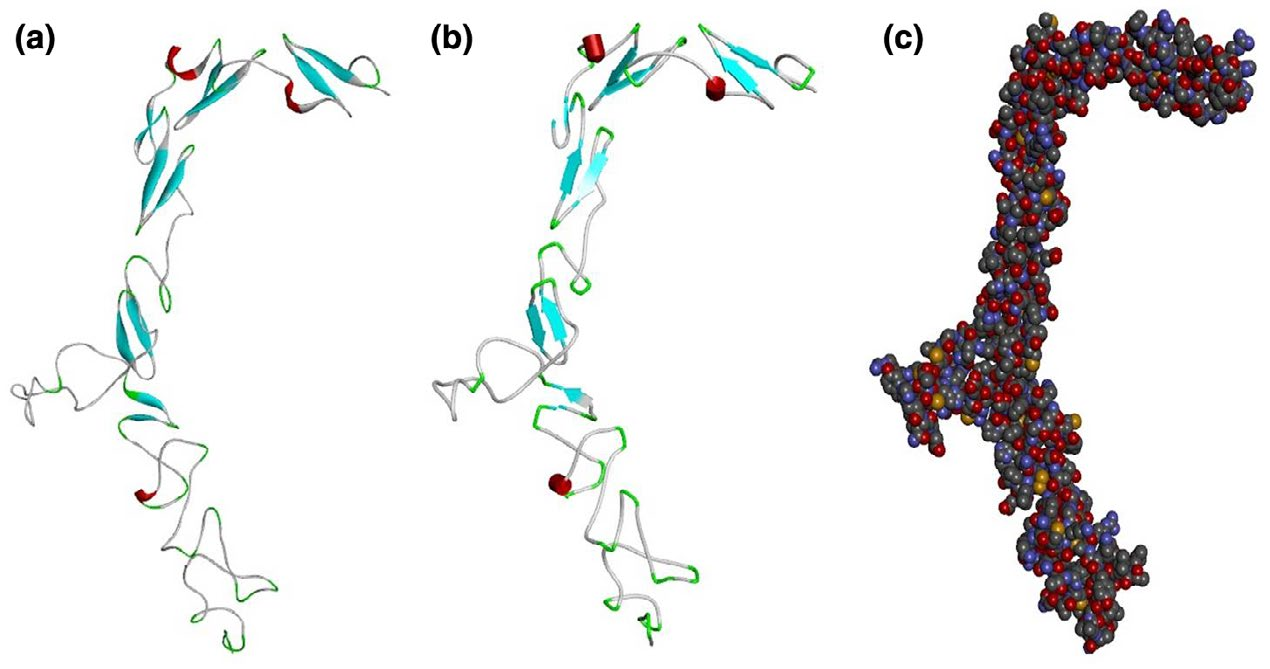
Modeled structure of the *NOTCH1* gene in (a) ribbon (b) schematic and (c) CPK view. Cyan, white and red indicate beta sheets, loops, and alpha helices, respectively. CPK view: carbon is shown in grey, oxygen in red, nitrogen blue, hydrogen white, chlorine green, sulfur yellow, phosphorus orange, iron reddish brown

**FIGURE 3:**
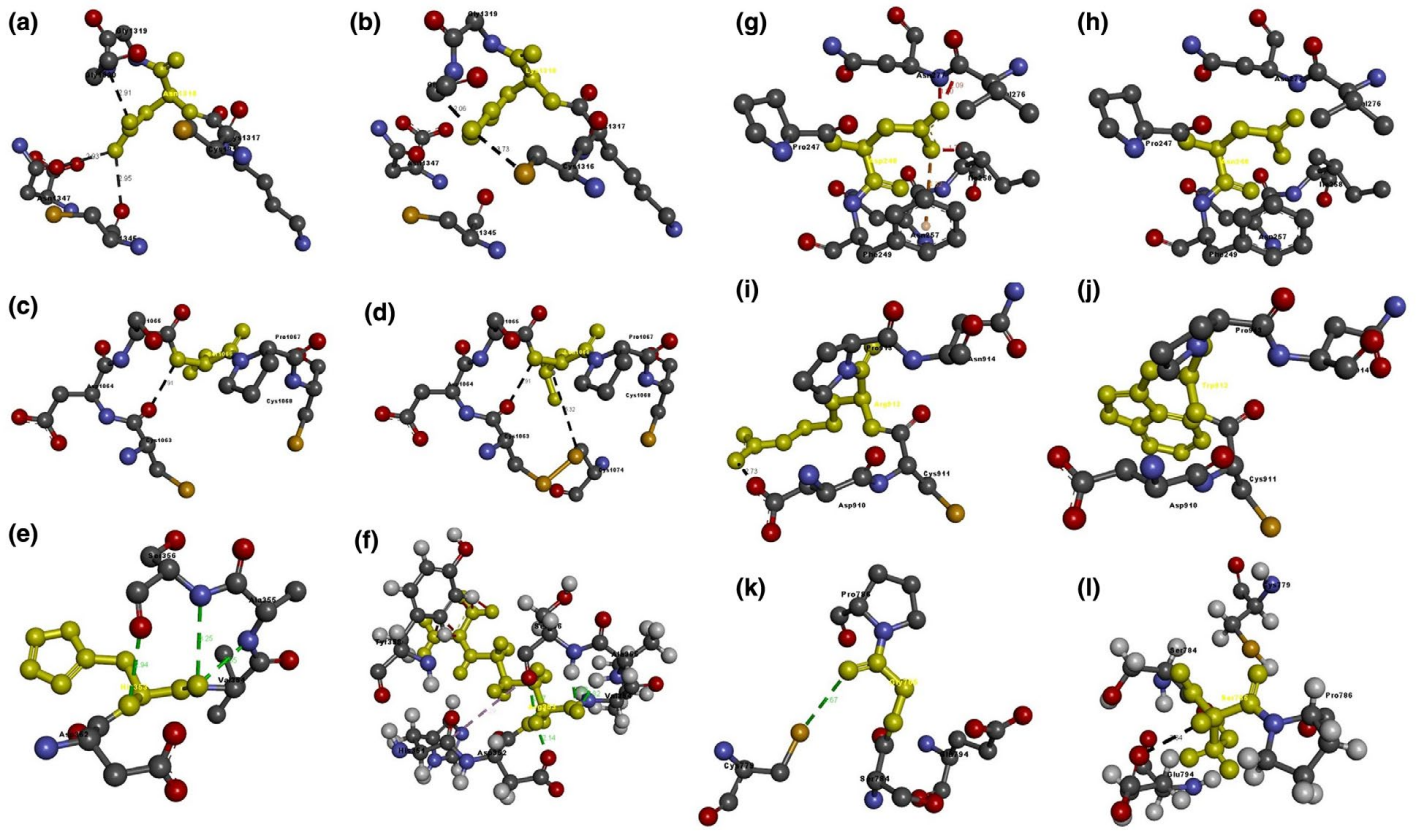
NOTCH1 protein model. Molecular interactions of (a) Asn1318 and (b) Lys1318 (in yellow) with adjacent residues. Molecular interactions of (c) Ser1066 and (d) Leu1066 (in yellow) with adjacent residues. Molecular interactions of (e) His353 and (f) Arg353 (in yellow) with adjacent residues. Molecular interactions of (g) Asp238 and (h) Asn238 (in yellow) with adjacent residues. Molecular interactions of (i) Arg912 and (j) Trp912 (in yellow) with adjacent residues. Molecular interactions of (k) Gly785 and (l) Ser785 (in yellow) with adjacent residues

Based on the percentage of sequence identity, similarity and best quality square, the 6py8.2.E chain was selected to align the template and query sequences for model building. The model is shown in Figure 2. We then used the Discovery studio visualizer to generate Asp238Asn, His353Arg, Arg912Trp, Ser1066Leu, and Asn1318Lys structural variants. Molecular‐level interaction analysis was performed between native/variant and adjacent residues. Snapshots are shown in Figure 3. Details of the residues involved in interactions along with the types of bond they form and bond lengths in angstrom units are listed in Tables 5, 6, 7, 8, 9, 10.

**TABLE 5:** Details of molecular interactions of (a) Asn1318 and (b) Lys1318 with adjacent residues in the NOTCH1 protein model

S.No	Mutation	Amino acid	Molecular interactions	Bond length in angstroms	Bond type
	Asn1318Lys	Asn1318	Asn1318:N—Cys1345:O	2.95	H‐bond
			Asn1318:N—Asn1347:O	2.93	H‐bond
			Gly1320:N—Asn1318:O	2.91	H‐bond
		Lys1318	Lys1318:N—Gly1320:C	2.06	H‐bond
			Lys1318:N—Cys1316:S	3.73	H‐bond

**TABLE 6:** Details of molecular interactions of (a) Ser1066 and (b) Leu1066 with adjacent residues in the NOTCH1 protein model

S.No	Mutation	Amino acid	Molecular interactions	Bond length in angstroms	Bond type
	Ser1066Leu	Ser1066	Ser1066:N—Cys1063:O	2.91	H‐bond
		Leu1066	Ser1066:N—Cys1063:O	2.91	H‐bond
			Cys1074—Leu1066	5.32	H‐bond

**TABLE 7:** Details of molecular interactions of (a) His353 and (b) Arg353 with adjacent residues in the NOTCH1 protein model

S.No	Mutation	Amino acid	Molecular interactions	Bond length in angstroms	Bond type
	His353Arg	His353	His353 H: Asp352 O	2.14	H‐bond
			His353 H: Ser356 O	2.37	H‐bond
			His353 O: Ala355 H	1.22	H‐bond
			His353 O: Ser356 H	2.20	H‐bond
		Arg353	Arg353:C—Tyr358:C	2.16	H‐bond
			Arg353:C‐ Tyr358:H	1.36	H‐bond
			Arg353:N—Tyr358:C	1.99	H‐bond
			Arg353:N—Tyr358:H	1.20	H‐bond
			Arg353:N—Tyr358:C	2.27	H‐bond
			Arg353:N—Tyr358:H	1.58	H‐bond
			Arg353:H—Tyr358:H	1.45	H‐bond
			Arg353:H—Tyr358:C	1.94	H‐bond
			Arg353:H—:Tyr358:H	1.33	H‐bond
			Arg353:H—Ser356:O	2.37	H‐bond
			Ala355:H—Arg353:O	1.82	H‐bond
			Ser356:H—Arg353:O	2.20	H‐bond
			His351—Arg353	5.20	H‐bond

**TABLE 8:** Details of molecular interactions of (a) Asp238 and (b) Asn238 with adjacent residues in the NOTCH1 protein model

S.No	Mutation	Amino acid	Molecular interactions	Bond length in angstroms	Bond type
	Asp238Asn	Asp238	Asp248 O: Val276 C	2.09	H‐bond
			Asp248 O: Asn277 N	1.80	H‐bond
			Asp248 O: Ile258 C	1.71	H‐bond
			Asp248 O: Phe249 O	4.32	Pi‐interaction
			Asp248 O: Asn277 N	1.80	H‐bond
			Asp248 O: Val276 C	1.71	H‐bond
			Asp248 O: Phe249 O	4.32	Pi‐interaction
		Asn238	No bonds	–	–

**TABLE 9:** Details of molecular interactions of (a) Arg912 and (b) TRP912 with adjacent residues in the NOTCH1 protein model

S.No	Mutation	Amino acid	Molecular interactions	Bond length in angstroms	Bond type
	Arg912Trp	Arg912	Arg912 N—Asp910 O	2.73	H‐bond
		Trp912	No bonds	–	–

**TABLE 10:** Details of molecular interactions of (a) Gly785 and (b) Ser785 with adjacent residues in the NOTCH1 protein model

S.No	Mutation	Amino acid	Molecular interactions	Bond length in angstroms	Bond type
	GLy785Ser	GLy785	Gly785 O—Cys779 S	3.67	H‐bond
		Ser785	Ser785 H—Glu794 O	2.84	H‐bond

## 4 DISCUSSION AND CONCLUSIONS

From a cohort of 235 probands who were negative in our first‐tier genetic test, we identified seven patients having a lymphedema phenotype that carried rare missense variants in *NOTCH1*. This was carried out by NGS, including *NOTCH1* in the panel of genes screened. We also conducted a search of the literature for possible links between the role of *NOTCH1* and lymphedema. Mutations in the *NOTCH1* gene were shown to be associated with alterations of the lymphatic system in mouse models, resulting in the defective development of the lymphatic system and abnormal lymphangiogenesis. Blockade of Notch1 signaling, with specific function‐blocking antibodies, leads to defective postnatal lymphatic development in mice (Niessen et al., 2011). Moreover, it is known that LTB4 antagonism reversed edema and improved lymphatic function. *Notch1*‐/‐ mice were refractory to the beneficial effects of LTB4 antagonism, suggesting that LTB4 suppression of Notch signaling is an important mechanism in disease maintenance (Tian et al., 2017). In humans, variants in NOTCH1 are associated with autosomal dominant Aortic valve disease, Adams‐Oliver syndrome, and Tetralogy of Fallot (Garg et al., 2005; (Table 11). To our knowledge, no Genome‐Wide Association Study was carried out to link variants in *NOTCH1* with lymphedema. We also performed in silico analysis to compare molecular interactions between the wild‐type and variant amino acids and adjacent residues.

**TABLE 11:** Mouse and human phenotypes associated with *NOTCH1*

Gene	Function	OMIM Gene	Mouse model	Lymphatic phenotype	Human Phenotype
*NOTCH1*	Receptor involved in the development of many types of cells and tissues	190198	Key regulator of LEC sprouting and growth during lymphatic vessel morphogenesis in the developing mouse embryo (Fatima et al., 2014; Niessen et al., 2011; Zheng et al., 2011)	Defective postnatal lymphatic development in mice; pathological lymphangiogenesis(Niessen et al., 2011)	Adams‐Oliver syndrome 5, (616028) AD; Aortic valve disease 1, (109730) AD; Tetralogy of Fallot (Page et al., 2019)

Abbreviations: AD, autosomal dominant; LECs, lymphatic endothelial cells.

Our results show that three of seven probands are sporadic, while the other four cases carried the variant in family members. We performed family segregation studies and looked for correlations between genotype and phenotype, studying lymphatic alterations.

The first proband has sporadic edema of the lower limbs and lipedema, no family member performed genetic tests. Due to the lack of the template in some regions, we were unable to perform the Thr2132Met model.

The second proband has sporadic edema of the lower limbs and scrotum, no family member was available to perform genetic tests. In silico analysis of Asn1318Lys was performed and Asn1318 showed three interactions and the variant Lys1318 only showed two which are quite different from each other.

The third proband has sporadic edema of the right lower limb. His father has intermittent edema and segregates the same variant of the proband. For Ser1066Leu, there were no major differences in direct hydrogen bonding, but the variant Leu1066 showed one more interaction with Cys1074 which does not occur among the Ser1066 interactions.

The fourth proband has familial edema of the lower limbs and hands. Her sister and daughter segregate the same variant and have edema after lymphangitis and tonsillitis, respectively. In the case of His353Arg, His353 showed four interactions whereas the variant Arg353 showed 13 interactions, all quite different from the His353 interactions.

The fifth proband has sporadic edema of right lower and her mother carried the same variant. The mother subclinical tests show that she has cyclic edema in the lower limbs. In silico analysis showed that *NOTCH1* gene structure with Asp238 has major differences in stability with respect to the variant Asn238, the former showing seven interactions and the latter none.

The sixth proband has sporadic edema of the left lower limb. Her daughter and son carry the same variant. Both reported intermittent edema of the lower legs at the subclinical test. In the case of Arg912Trp, Arg912 showing one interaction and Trp912 none.

The seventh proband has sporadic right lower limb edema. No family member was tested, bet her clinical history reported that a cousin of the mother is affected. In the case of Glu785Ser, Glu785 showed one interaction, whereas the variant Ser785 showed another, different from the original.

In conclusion, these findings suggest that the overall protein structure is somehow altered by these different interactions, leading to functional defects in the protein. Our findings support the hypothesis that variants of *NOTCH1* could predispose our patients to lymphedema and that *NOTCH1* should be considered as a novel candidate gene for lymphedema in humans.

## DISCLOSURE STATEMENT

No competing financial interests exist.

## AUTHOR CONTRIBUTION

SM enrolled the patients, performed the clinical evaluation, and wrote the manuscript; MR, RS enrolled the patients and performed the clinical evaluation; SB performed the genetic analysis and wrote the manuscript; SK, DV wrote the first draft; DK, MB, AD, MD, JK critically revised the manuscript; SHB, SP performed the bioinformatic analysis and wrote the relevant section in the manuscript; MB supervised the work and critically revised the manuscript.
